# Improving homology modeling from low-sequence identity templates in Rosetta: A case study in GPCRs

**DOI:** 10.1371/journal.pcbi.1007597

**Published:** 2020-10-28

**Authors:** Brian Joseph Bender, Brennica Marlow, Jens Meiler

**Affiliations:** 1 Department of Pharmacology, Department of Chemistry, and Center for Structural Biology, Vanderbilt University, Nashville, Tennessee, United States of America; 2 Institute for Drug Discovery, Leipzig University Medical School, Leipzig, SAC, Germany; University of Virginia, UNITED STATES

## Abstract

As sequencing methodologies continue to advance, the availability of protein sequences far outpaces the ability of structure determination. Homology modeling is used to bridge this gap but relies on high-identity templates for accurate model building. G-protein coupled receptors (GPCRs) represent a significant target class for pharmaceutical therapies in which homology modeling could fill the knowledge gap for structure-based drug design. To date, only about 17% of druggable GPCRs have had their structures characterized at atomic resolution. However, modeling of the remaining 83% is hindered by the low sequence identity between receptors. Here we test key inputs in the model building process using GPCRs as a focus to improve the pipeline in two critical ways: Firstly, we use a blended sequence- and structure-based alignment that accounts for structure conservation in loop regions. Secondly, by merging multiple template structures into one comparative model, the best possible template for every region of a target can be used expanding the conformational space sampled in a meaningful way. This optimization allows for accurate modeling of receptors using templates as low as 20% sequence identity, which accounts for nearly the entire druggable space of GPCRs. A model database of all non-odorant GPCRs is made available at www.rosettagpcr.org. Additionally, all protocols are made available with insights into modifications that may improve accuracy at new targets.

## Author summary

Structure-based drug discovery is among the new technologies driving the development of next generation therapeutics. Inherent to this process is the availability of a protein structure for virtual screening. The most heavily drugged protein family, G-protein coupled receptors (GPCRs), suffers from a lack of experimental structures that could hinder drug development. Technical challenges prevent the determination of every protein structure, so we turn to computational modeling to predict the structures of the remaining proteins. Traditional techniques fail due to low sequence identity of available templates. Here, we test improvements to model building and identify changes that can improve accuracy of modeling from templates below 40% identity. We apply our findings to model building of all non-odorant GPCRs to build the RosettaGPCR database (www.rosettagpcr.org) for use in future drug development.

## Introduction

### G-protein coupled receptors represent important therapeutic targets

G-protein coupled receptors (GPCRs) are the largest family of membrane proteins in the human body comprising nearly 800 distinct receptors [1]. They orchestrate cellular responses to extracellular signals and thus play roles in immune function, cardiopathies, and neural development. They are a ubiquitous family of proteins evolved over time to respond to a variety of stimuli including ions, small molecules, larger peptides, and even light [2]. Given their expression on the cell membrane, they are attractive targets for therapeutic intervention. Current estimates suggest around 30% of approved drugs act at a GPCR [3].

### Experimental structures of GPCRs are determined at an increasing rate overcoming substantial obstacles

The first atomic resolution structure of a GPCR was rhodopsin in 2000, in part due to its high abundance and stability from native sources [4]. For most receptors, expression levels are well below what is needed for structural characterization from orthologous sources. Therefore, it wasn’t until 2007 that the structure of a second receptor was experimentally determined [5,6]. As dynamic, membrane-bound proteins, significant protein engineering was needed for structure determination (i.e. thermostabilization through mutation, nanobodies, fusion proteins, and/or truncation of flexible termini) [7]. Since 2007, over 50 unique receptor structures have been determined. While this is a tremendous achievement, this represents only about 6% of the GPCR superfamily. Even when focusing on non-olfactory GPCRs that are generally considered as druggable targets, nearly 350 unique receptors remain to be structurally characterized either for better understanding of how current drugs bind their targets or for structure-based drug discovery. Of importance, at least 100 non-olfactory GPCRs have been designated orphan receptors due to a lack of chemical matter [8]. Knowledge of the structural details of the ligand binding pocket could assist in identifying chemical probes for these dark receptors.

### Computational modeling can extend our current understanding of GPCR structures

Given this knowledge gap, homology modeling is an important tool for generating models of as-of-yet undetermined protein structures. Homology modeling uses a protein template with a shared topology to map the target sequence onto its backbone coordinates in a process called threading [9]. Early homology modeling relied on a single template structure for target structure prediction. However, these methods fail to generate accurate models using templates with low sequence identity to the target protein. More recently, the use of multiple templates has seen success in modeling targets in which the sequence identity is below 50% to any given template [10,11]. Given that GPCRs often share identities in the range of 20–30%, GPCR-specific model building has largely moved towards multiple template homology modeling. Servers for the prediction of GPCRs from multiple templates are available including GPCR-ModSim [12], GPCR-I-Tasser [13], GPCRM [14], GPCR-SSFE [15], and GPCRdb [16]. GoMoDo, another server, uses single-template modeling [17]. The underlying software for all these servers is Modeller [18], except for GPCR-I-Tasser [11]. To date, no GPCR-specific multi-template modeling method has been developed in Rosetta, a software unique in its capability to not only predict structures but also design new functionality and dock ligands among other applications [19,20]. Despite a unified method for GPCR modeling, Rosetta’s performance on single-template modeling of GPCRs has been analyzed in the past with some success. In the GPCR Dock experiment [21], Rosetta performed best in the structure prediction of the Smoothened receptor ligand binding pose [22].

### Rosetta hybridizes multiple templates

While other methods predefine template segments for various parts of the target model or averages template structures, Rosetta handles multiple templates simultaneously during its modeling process [23]. Rosetta holds all templates in a defined global geometry and randomly swaps parts of each template using Monte Carlo sampling to identify regions from the various templates that best satisfy the local sequence requirements. This template swapping occurs in parallel with the traditional peptide fragment swapping from a database derived from the PDB based on the target sequence and predicted secondary structure, a hallmark of Rosetta’s folding algorithm [24]. This simultaneous sampling of template segments and peptide fragments allows the energy function to define which segments to keep from the various templates based on how well each segment improves the overall score of the model. Hybridization of templates has been shown to be successful in CASP experiments, particularly for low template identity targets down to 40% [23]. Below 40% identity, Rosetta is capable of producing accurate models, though it is not known *a priori* if the output models will be reliable.

### Improving the protocol for modeling of targets with identity below 40%

Given the past success of Rosetta in single template homology modeling of GPCRs [22] and the novel strategy of multiple template modeling in the Rosetta framework [23], we set out to optimize the protocol for low identity template modeling with a focus on the therapeutically relevant GPCR family. The change from the previous single-template homology modeling to multiple-template modeling was multifaceted and we tested each component individually. First, the use of multiple templates begs the question of the optimal number of templates to use. Previous work in multiple template homology modeling suggested that there is a goldilocks effect in which multiple templates are better than one but too many templates could actually hurt the modeling process [25]. Additionally, as Rosetta uses a peptide fragment library, we evaluate its influence on enhancing model accuracy. Further, we now handle loop closure simultaneously in Rosetta’s multiple-template homology modeling through the use of these peptide fragments. As loops are often involved in ligand- or protein-protein recognition, we optimized the alignment in these regions and tested its effect on model accuracy. We benchmarked our methods on a subset of 34 available structures of unique GPCRs covering the four classes (A, B, C, and F). Additionally, we chose to model all targets using templates below 40% sequence identity, unless otherwise noted, to mimic the situation when predicting novel target structures. We find that our improvements result in highly accurate models due to the curated sequence alignments and peptide fragment utilization. This improved method can now accurately model Class A receptors down to a template identity of 20%. Based on this success, we established a database of input code and output models for all human non-odorant receptors available for public use (www.rosettagpcr.org). While we focus on GPCRs for this benchmark, we emphasize that these modifications can assist in the modeling of any target from templates with low sequence identity.

## Methods

### Description of benchmark data set

For this study we chose a subset of 34 crystal structures of GPCRs covering the four structurally characterized classes A, B, C, and F. In total there were 29 Class A receptors, two Class B receptors, two Class C receptors, and one Class F receptor (S1 Table). Importantly, we chose to model the receptors using exclusively templates below 40% sequence identity, unless explicitly noted, as this most closely resembles the majority of real-life cases when modeling GPCRs.

### Generation of multiple-sequence alignments (MSA) of templates

Initial alignments for the benchmark set were obtained from the GPCRdb [26]. This largely ensured that the transmembrane α-helices were well aligned. To improve on these alignments, the structures were aligned and visualized in PyMol, and the structural alignments were compared to the sequence alignments. Transmembrane helical sequences were aligned starting from the most conserved residue in each α-helix and extended outwards using the structural alignments to guide insertion and deletions along the α-helical axis. Loop alignments were generated based on the alignment of vectors of Cα to Cβ atoms between receptor structures. If secondary structural elements were present in loop regions such as disulfides, α-helices, or β-sheets, these were preserved in the alignment. Remaining residues that could not be aligned by any of the above metrics were moved to be adjacent to a region of defined secondary structure to ensure proper fitting of peptide fragments between ordered and unordered regions. The alignment of the 34 receptors is shown in S1 Fig and are available online. Additional alignments for comparison were generated using the default options of ClustalOmega [27], Muscle [28], T-Coffee TM-PSI and Espresso [29], and Mustang [30] and used without further modification.

### Template selection

For all receptors, a pairwise identity matrix covering the transmembrane bundle and loops and excluding long termini was generated using ClustalOmega [27]. The reported identities were used to rank the templates for each receptor model. Shown in S2 Table is the ranked list of templates for each target receptor in the benchmark. While most templates have sequence identities below 40%, those highlighted in yellow were removed because they featured sequence identity above the 40% threshold. Templates labeled in bold were used in single-template high identity modeling to compare to the previous benchmark [22].

### Generation of additional input files

Membrane spanning topology files were generated by submitting the sequence of the target proteins to Octopus [31]. The output files were converted into Rosetta readable span files with Rosetta’s built in octopus2span.pl script. Disulfide bond restraint files were prepared for each target protein for the conserved disulfide bond between TM3 and ECL2, except for LPA1 and S1P1. Additional disulfide bonds within ECL3 were mapped as needed.

### Sequence alignment of target sequence to template MSA

Alignment of sequences without known structure was accomplished similarly as above. First, alignments were extracted from GPCRdb. Then the highly conserved residues in each helix were aligned with the template MSA. Positioning of residue x.50 (BW numbering) often corrected helix alignments but gaps and deletions were propagated throughout families. For receptors lacking the highest conserved residue in each helix, other motifs such as DRY, NPxxY, and CxxP were used for helix positioning. The loops were aligned as such: ECL1, ICL1, and ICL2 were aligned using common sequence motifs (eg. xWxxG in ECL1). For ICL3, sequence alignments were maintained within families particularly with receptors with short loops such as the Class B and chemokine receptors. For the majority of receptors, the ICL3 sequence was split at the halfway point between TM5 and TM6 and the halves were adjoined directly to the end of TM5 or beginning of TM6, respectively. For ECL3, particular attention was given to the presence of cysteines for either internal ECL3 disulfides or disulfides between the N-terminus and ECL3. These cysteine residues were used for alignment. For receptors lacking disulfide bonds, patterns identified in template families were used to fix family alignments. Remaining receptors again had the sequence of the loop halved and adjoined the halves to their next helical sequence for peptide insertion overlap. For ECL2, targets were grouped by putative ligand binding type: i.e. aminergic, lipid, peptide, unknown. Based on this grouping, the alignments were carried out specifically for their family type (i.e. a beta sheet was predicted and aligned for all peptide receptors). For receptors with unknown ligand type or dissimilar ligands (i.e. protons), the loop sequence was first divided at the conserved cysteine residue and this residue was aligned generating two shorter loops. These loops were then halved and adjoined to their nearest fixed structural feature, either a transmembrane helix or the conserved disulfide bond with TM 3. The full MSA of all receptors is available at www.rosettagpcr.org and www.github.com/benderb1/rosettagpcr.

### Model production

With all input files in hand, target sequences were threaded onto the pre-aligned templates using Rosetta’s partial_thread application [23]. Threaded models were passed to the hybridization application via use of Rosetta XML scripts [23,32]. Either 100 models or 1000 models were generated per run as noted in the text.

## Results

### Blended sequence- and structure-based alignment is critical for modeling success

Inherent to any homology modeling protocol is an alignment between the sequence of the target protein and the template structure. This alignment maps the target sequence onto the template structure in a process called threading [9]. Sequence alignments are necessary for this process, and a wide variety of search methods have been generated [27,28]. Each sequence alignment method uses a different algorithm to weight the importance of sequence conservation globally or locally with or without gap penalties. As we learn more about the structures of diverse proteins, it becomes apparent that structure is often better conserved than sequence. As such, additional algorithms have been generated based on structural alignments and domain fold recognition [29,30]. This latter case is inherent to the family of GPCRs in which the common sequence identity between receptors is around 30% while all receptors share a similar structural fold. The best-known alignment of GPCRs is Ballesteros-Weinstein (BW) numbering [33] which identifies the most conserved residue in each α-helix and sets it as a starting point for alignment (i.e. 3.50 being the most conserved residue in helix 3). Counting along the α-helix in reference to this residue all other residues are enumerated. While useful, this alignment falls short in two areas. As more receptor structures became available, it was found that not all receptor families adhere strictly to the *i* to *i+4* periodicity in every α-helix [34]. Insertions and deletions have resulted in local alterations of the helicity, in particular around proline or glycine residues. Secondly, BW numbering fails in the loop regions as different receptors have varying lengths of α-helices and dramatically different loop structures particularly within extracellular loop 2 (ECL2), which can adopt disordered conformations, α-helices, or β-sheets. As these loops are critical for ligand recognition [35], they diverge widely in sequence proving even more challenging for the creation of meaningful sequence alignments. However, despite sequence divergence, there is evidence for structural conservation in these regions [36]. Therefore, a critical component to the present method has been to blend sequence and structure information into an optimized knowledge-based sequence alignment for the templates with an emphasis on structural alignments in the loop regions (S1 Fig and Methods).

**Fig 1:**
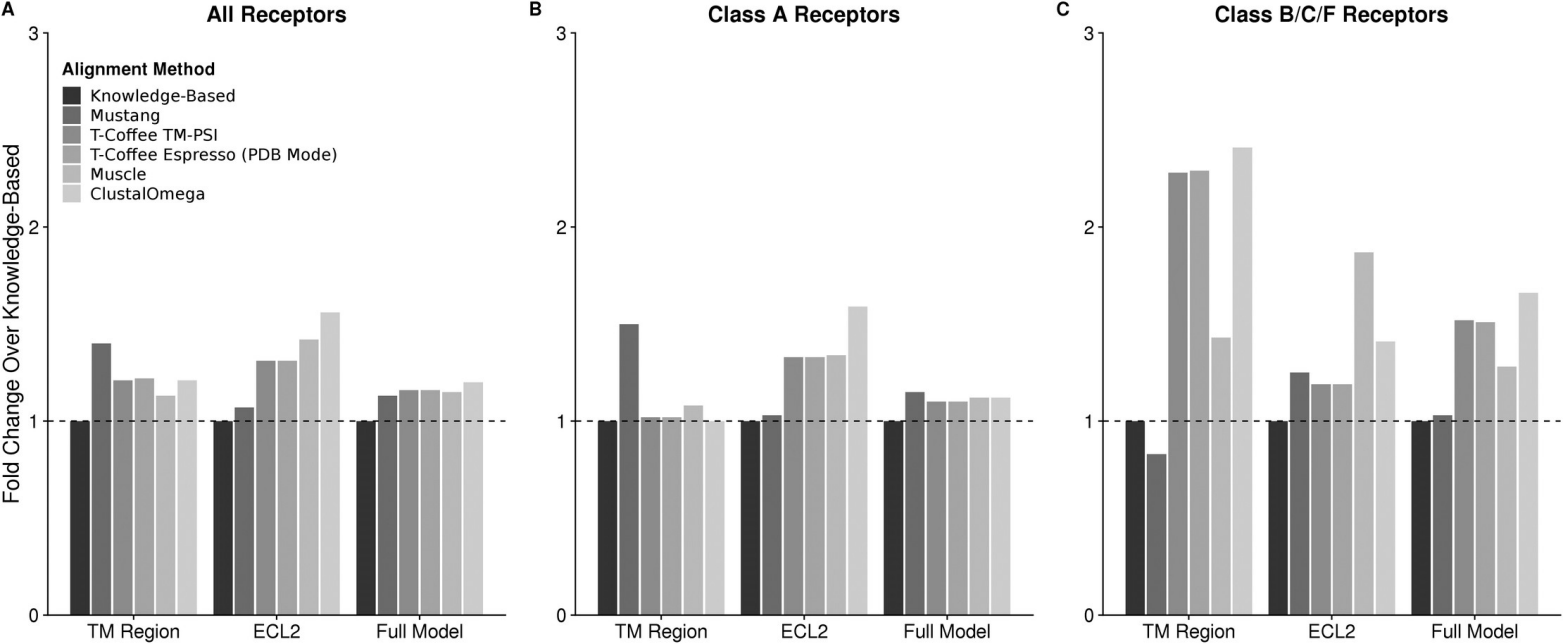
Comparison of average RMSD change using various alignment methods. A total of 100 models were produced for each alignment method. The average RMSD of the models were normalized to the average RMSD of the models produced with the knowledge-based alignment (black). Values above 1 represent an alignment method that produced on average worse models while values below 1 represent an alignment method that produced on average better models. For (A) all receptors regardless of family, the knowledge-based modeling performs the best regardless of region analyzed. When split between (B) Class A and (C) Classes B, C, and F, the majority of the improvements are found in the Classes B, C, and F where template availability is limited.

We compared this new alignment to other sequence- or structure-based alignment methods. For each receptor in the benchmark, 100 models were generated for each of the six alignment methods tested. The average RMSD for a target protein was divided by the average RMSD for the same target using the new alignment resulting in a fold change and the average across the full benchmark is reported (Fig 1). As seen, despite using sequence-only (ClustalOmega [27] and Muscle [28]), structure-only (Mustang [30]), or automated blended alignments (T-Coffee, TM-PSI, and Espresso (PDB Mode) [29]), the knowledge-based alignment performs the best in all regions tested. For Class A receptors it is found that the transmembrane (TM) region is modeled nearly equivalently across all methods. However, since the overall accuracy was improved, we sought to identify which non-transmembrane component was contributing to this improvement. Incredibly, we find that ECL2, the longest of the extracellular loops and most difficult for modeling, was dramatically improved by our alignments. We find this to also be true for the Class B, C, and F receptors. Though in those families, TM accuracy also improves. Altogether, this demonstrates that a blended sequence- and structure-based alignment can improve accuracies across both structurally defined regions (TM) and long, challenging loops (ECL2). This has implications for other systems in which loops can be critical for protein function and binding partner recognition. In particular this is analogous to research in antibody modeling in which protocols have been developed to model the long loops of the complementary determining regions [37,38].

### Peptide fragment hybridization improves target model quality

**Fig 2:**
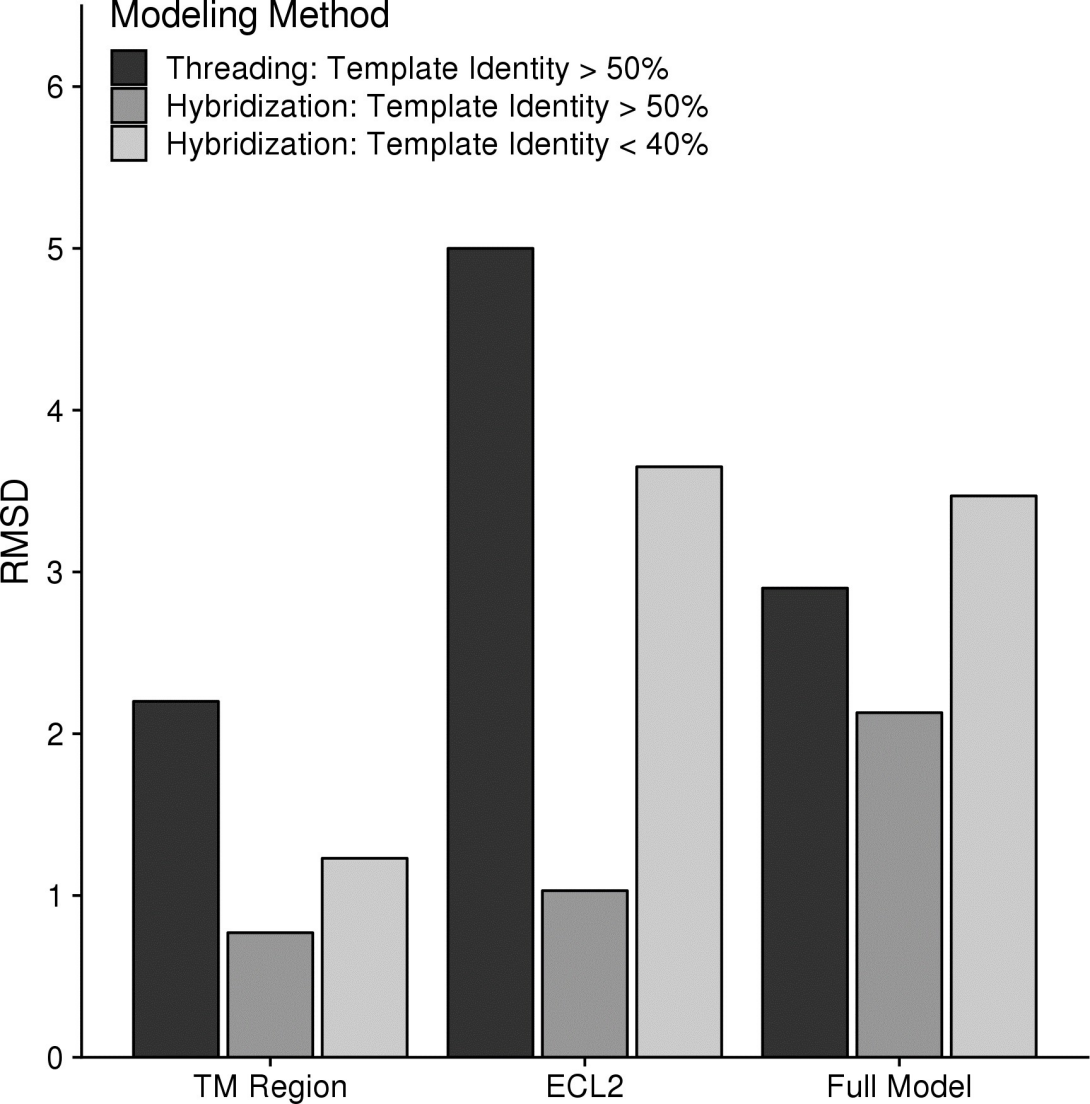
Comparison of single template modeling methods with peptide insertion. Using only a subset of receptors and templates that were available in our original GPCR modeling benchmark (yellow in S2 Table), 100 models were generated using either a single high identity template or the best template available below 40%. Original results from the 2013 benchmark [22] are displayed in black. Using the hybridize code with the same original templates dramatically improved the results across all measures (medium grey). Using a low identity template in hybridize (light grey) expectedly worsened the results compared to the high identity template but was either better or comparable with the original threading alone algorithm.

Our previous benchmark of GPCR modeling relied on single-template threading [22]. We wanted to recapitulate this initial study using the Hybridize code [23] to allow for peptide fragment insertion to see what effect peptide sampling had on output quality. Peptide fragments are mined from the PDB based on the target sequence and can improve local geometries that may be inaccessible from template structures [24]. This analysis was limited to the eight receptors with high identity templates available in the 2013 benchmark (β1AR, β2AR, M2R, M3R, δOR, κOR, μOR, and NOPFQ). In this experiment, each target was modeled on the single best available template with sequence identity either greater than 40% or less than 40% and allowed to hybridize with the peptide fragment library (S2 Table). As seen in Fig 2, using the exact same template as was used in the previous threading-alone method, hybridization of template structures with peptide fragments can substantially improve output model accuracy in all measured regions. The transmembrane region improves on average by more than one Angstrom to 0.8 Å root-mean-square deviation (RMSD) to the crystal structures showing highly accurate modeling of this region. The ECL2 region again showed a dramatic improvement with an average RMSD to the crystal structures of 1.0 Å compared to the previous method of single-template modeling without peptide fragment insertion that reported an average RMSD of 5.0 Å. The full model RMSD, which accounts for all remaining loops and flexible termini, also showed modest improvement from 2.9 to 2.1 Å. These results were similar when using a single template with sequence identity less than 40%. Both the TM region and ECL2 improved by at least 1.0 Å while the Full Model RMSD actually worsened by 0.5 Å. This is likely due to the fact that divergent receptors have dramatically different termini conformations, which are often affected by crystal packing artifacts. Taken together, peptide insertion accounts for a substantial improvement over threading alone even when templates of sequence identity below 40% are used.

### Multiple templates improve performance for low sequence identity targets

**Fig 3:**
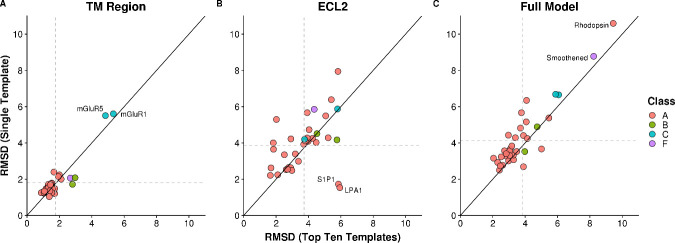
Comparison of average RMSDs for single versus multiple template homology modeling. Using either one template or ten templates, 1000 models were generated for each target and the average RMSD was calculated over the TM region (A), ECL2 (B), and the full model (C). Values that fall above the diagonal performed better when using multiple templates and values that fall below the diagonal performed better with a single template. Targets are colored by class.

**Fig 4:**
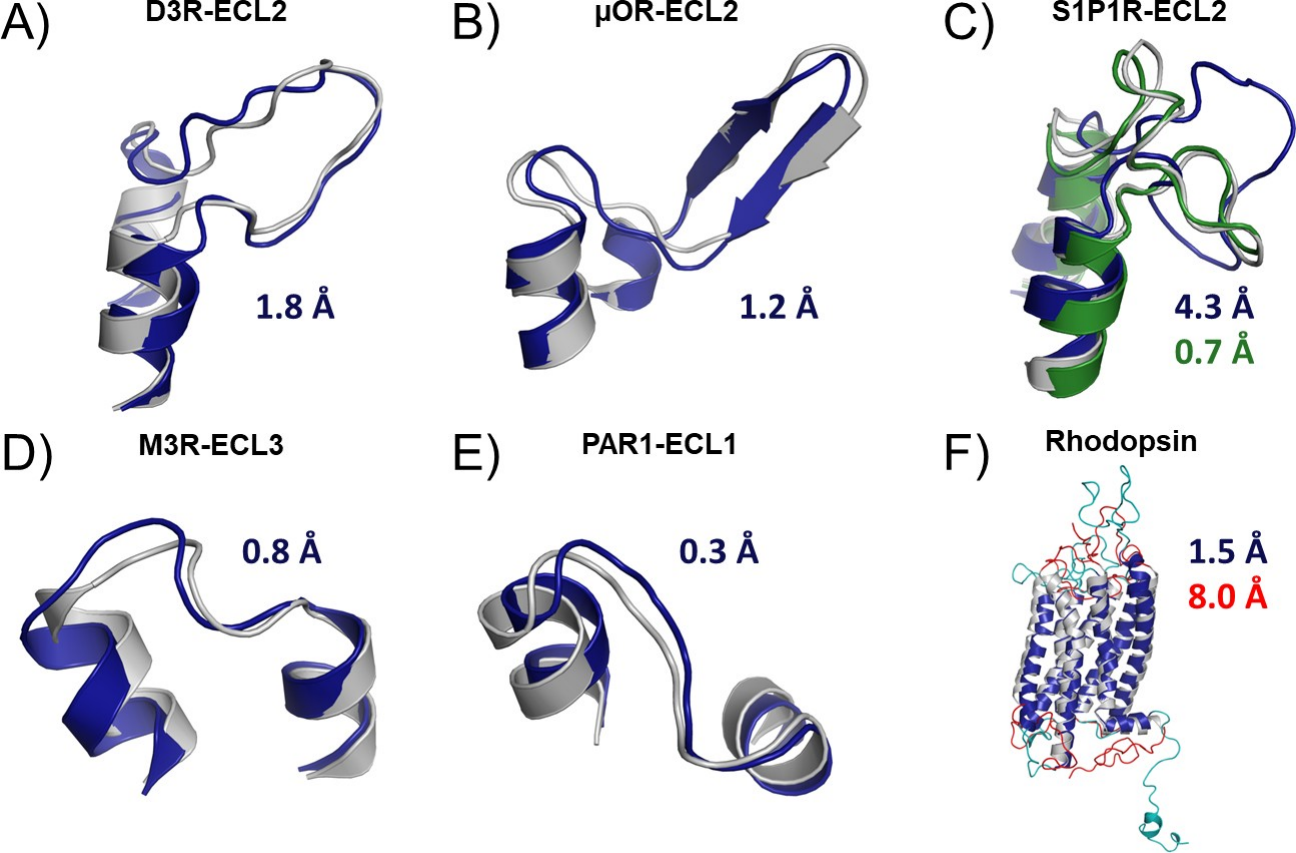
Examples of results obtainable with RosettaGPCR. In all cases, the crystal structure is colored grey and the model is blue. Three different ECL2 loops structures are presented: disordered (A), β-sheet (B), and lipid-binding (C). RosettaGPCR performs well on loops containing the conserved disulfide. For lipid receptors lacking the conserved disulfide (C) multiple templates (blue) perform worse than using a single template with similar structure (green), in this case the LPA receptor. Extracellular loops 3 (D) and 1 (E) also perform quite well with this method. In general, RosettaGPCR can model the TM region of most receptors below 2 Å (F). However, for receptors like rhodopsin with complex loop structures and termini (red), the model (cyan) fails to capture the overall conformation (8.0 Å RMSD).

While peptide insertion helped improve accuracy in the TM and ECL2 regions, overall model accuracy weakened when using a single template with sequence identity less than 40% to the target model. Therefore, we expected that multiple templates could overcome the shortcomings of any single template when modeling a target with low identity templates [23]. We generated 1000 models for every receptor in the benchmark using either the single best template less than 40% identity or the ten best available templates under 40% identity and compared the average RMSD of the resulting models (Figs 3 and 4). As expected, the average RMSDs improved for almost all receptors in the ECL2 and Full Model criteria. The TM region was rather insensitive to the increase in template availability showing on average only 0.05 Å improvement for the whole set. In both conditions, the majority of receptors are modeled with accuracies in this region below 2 Å. A few exceptions to the above trends deserve attention as they may influence target-specific modeling. In the TM region, both Class C receptors perform rather poorly with either set of templates. This is due to the fact that the 40% threshold for selecting templates removed the other Class C receptor from the template pool such that they were modeled with non-Class C templates, a feature we recommend against for general modeling. As the structure of the TM region is distinct for these proteins compared to the other classes, the error was expected to be high. In contrast, for the Class B receptors, the two structures have a sequence identity of 35% with respect to one another allowing these structures to be included as templates in the benchmark resulting in improved performance. In ECL2, there are two Class A receptors (S1P1 and LPA1) that perform extremely well when using a single template as compared to ten templates. These are the only two receptors in the benchmark that lack the conserved disulfide between ECL2 and TM3. Their loop structures are distinct from all other receptors and as a result, loop modeling only performs well when using the single related template (Fig 4C). In the full model RMSD, both Rhodopsin and the Smoothened receptor perform poorly regardless of the modeling method used. This is because both have extremely long and unusual loops and termini (Fig 4F). Of note, the TM regions of these two receptors are modeled to 1.5 Å and 2.7 Å RMSD to the crystal structures of 1U19 and 4JKV, respectively. Additionally, only two Class A receptors perform worse in the Full Model RMSD calculation when using multiple templates. These again are S1P1 and LPA1 which performed poorly in the ECL2 modeling. It appears that the poor quality of ECL2 is reflected in the Full Model RMSD as the difference in the TM region for these two structures is only 0.1–0.2 Å. These observations are important when deciding the best template set for a new target. If the loops and termini are distinct from any known template as in the case of Smoothened, it can be expected that this method will underperform. Further, if the loop sequences and predicted secondary structures are similar to only one available template, it would be expected that using a single template would be better than multiple templates.

### Identification of the optimal number of templates

**Fig 5:**
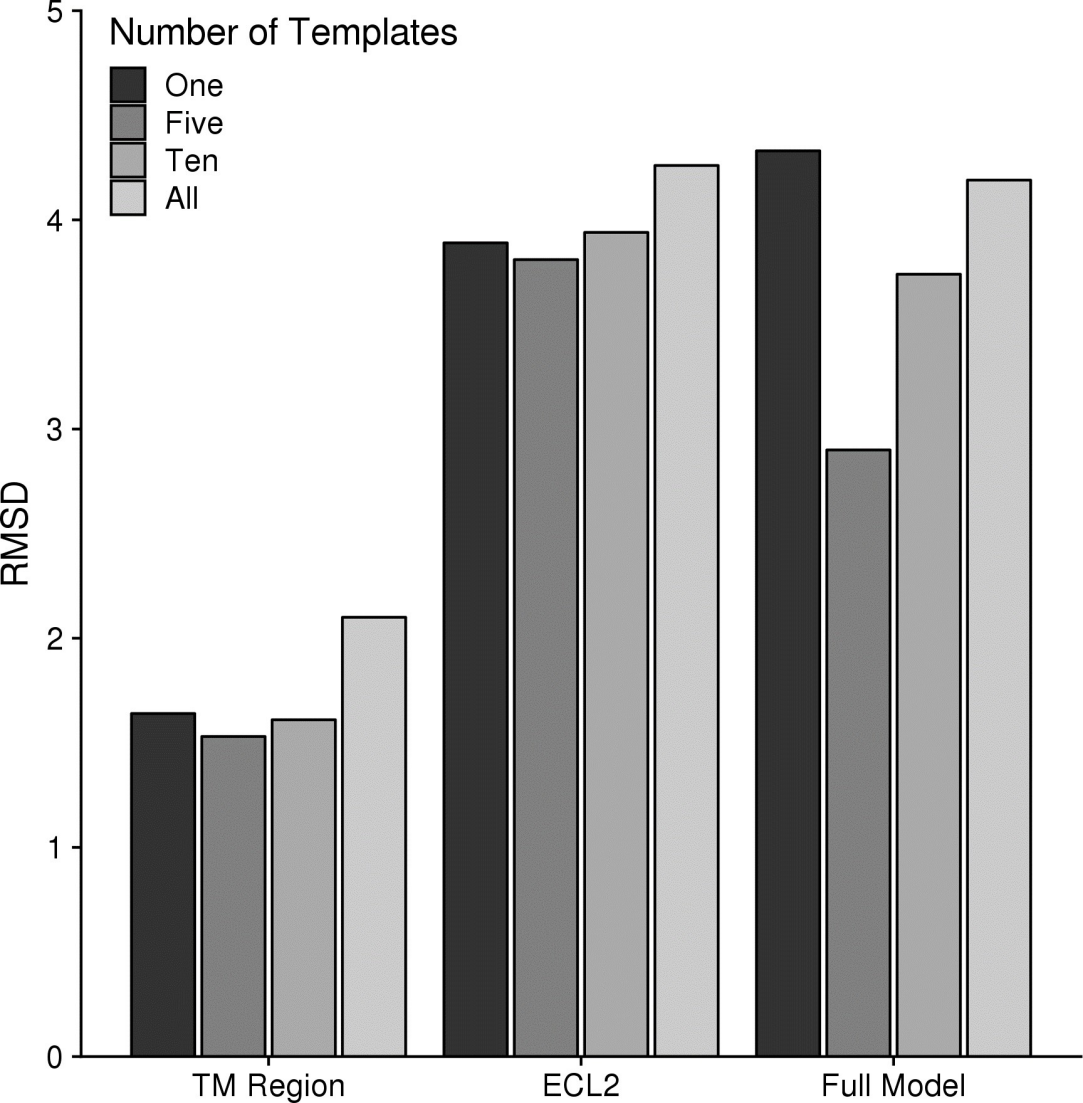
Comparison of model accuracy using various numbers of starting templates. For each target, 1000 models were generated using either 1, 5, 10, or all available templates. The average RMSD is plotted for the TM region, ECL2, and the Full Model.

As reported previously for multiple template homology modeling, the use of multiple templates, while improving results over the single template approach, will weaken model accuracy if too many templates are used [25]. Thus, we determined an optimal number of templates on average for GPCR modeling using our method. We generated an additional 1000 models for each receptor using either five or all available templates and compared the data with the previous data on one and ten templates (Fig 5). For both the TM region and ECL2, using all available templates was worse than any other set of templates while the average RMSDs were quite similar for one, five, and ten. However, for the full model accuracy, using a single template was worse than all other template sets. Five templates performed distinctly well in the full model accuracy compared to the other template sets while only providing modest improvement over the other template sets in the TM and ECL2 regions. Therefore, we suggest five templates to be the best number of templates on average for modeling GPCRs with our method. In comparison to the previous benchmark, the set of five templates was the only set of low identity templates that performs better than a single high identity template without hybridization over the full model (Fig 2). Again, this doesn’t take into account any of the exceptions mentioned above. When modeling any one receptor in particular, an expert user would want to use all available knowledge to determine the best template pool for modeling. Therefore, we provide all input files needed for modeling so that a user can optimize the process using their expert knowledge.

### The improved protocol is competitive with other GPCR modeling servers

We next compared our method with other GPCR modeling servers. Three servers with publicly available databases of GPCR models, GPCRdb [16], GPCR-I-Tasser [13], and GPCR-SSFE [15], were included. We identified four human GPCR structures that were released following the conclusion of method development. The four structures were C5aR1, Y1R, PTAFR, and D2R (PDB IDs 6C1R [39], 5ZBQ [40], 5ZKQ [41], and 6CM4 [42], respectively). Of note, the GPCR-SSFE database lacked a structure of the D2 receptor for comparison. We generated 100 models of each receptor target using five template structures and selected the best model by total energy. In comparing our model with the models generated by other servers, we find that RosettaGPCR consistently performs among the best of the predictions (Table 1). We find two cases of other servers outperforming our models. For the C5aR1 structure, GPCR-I-Tasser just barely achieves a better RMSD for the full model (1.649 vs 1.661 Å). GPCRdb built a better ECL2 of Y1R compared to ours with RMSDs of 1.617 Å versus 1.896 Å, respectively. We acknowledge these servers in many cases do well, though all can struggle with ECL2. However, the exact reason differences exist between modeling servers is multifaceted. While the underlying modeling algorithm differs (i.e. Modeller, I-Tasser, Rosetta), each server may also select different templates and importantly use different alignment methods. For example, GPCRdb only aligns helical segments of the receptors while largely ignoring loop alignments. In contrast, we find that manual curation enables a bridge between sequence- and structure-based alignments. Altogether, RosettaGPCR performs as well if not better for these test cases. Further, the strength in a Rosetta approach is the resulting models will be directly transferable to additional applications within the larger Rosetta suite including docking and design.

**Table 1:** Results of Novel Structure Prediction from Various GPCR Modeling Servers. Blind predictions were carried out on C5aR1 (PDB ID 6C1R [39]), Y1R (PDB ID 5ZBQ [40]), PTAFR (PDB ID 5ZKQ [41]), and D2R (PDB ID 6CM4 [42]). 100 models were generated for each target with RosettaGPCR and the best scoring model was used for analysis. The RMSD of each model for the various servers were calculated for the TM region, ECL2, and the full model. The best performing model in each evaluation criteria is bolded. No data is available for GPCR-SSFE for D2R.

Target	Region	RosettaGPCR	GPCRdb	GPCR-I-Tasser	GPCR-SSFE
C5aR 6C1R	TM	**1.233**	1.854	1.294	1.901
	ECL2	**1.606**	2.58	1.944	9.126
	Full Model	1.661	2.083	**1.649**	2.456
Y1R 5ZBQ	TM	**0.936**	1.765	1.271	1.132
	ECL2	1.896	**1.617**	2.339	9.464
	Full Model	**1.361**	2.296	1.704	2.297
PTAFR 5ZKQ	TM	**1.310**	1.559	1.800	1.355
	ECL2	**4.370**	4.658	5.174	9.535
	Full Model	**1.897**	2.181	2.872	2.679
D2R 6CM4	TM	**1.113**	1.485	1.604	--
	ECL2	**4.193**	6.011	4.431	--
	Full Model	**1.536**	2.318	2.304	--

### Accuracy of models with increasingly worse templates does not decline linearly

**Fig 6:**
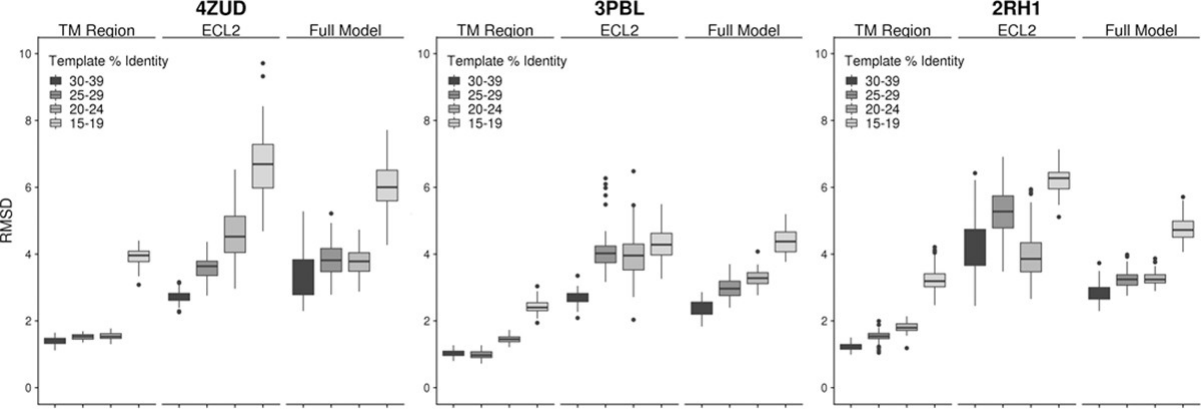
Model accuracy with templates over multiple sequence identity ranges. Three receptors (4ZUD [43], 3PBL [44], and 2RH1 [5]) were identified that had at least 5 templates in each identity range (30–39%, 25–29%, 20–24%, and 15–19%). Using the identified 5 templates for each identity range, 100 models were generated and the RMSD of these models is displayed in box and whisker plots for each region (TM, ECL2, and Full Model).

In our previous work on GPCR modeling using single-template threading, it was found that templates needed to have greater than 50% sequence identity for accurate models [22]. Subsequently, the use of multiple-template modeling in Rosetta was suggested to be accurate to about 40% sequence identity [23]. However, in this current benchmark we only use templates with less than 40% sequence identity and still produce highly accurate receptor models. Therefore, we wanted to identify a new lower threshold for template sequence identity. We devised an experiment where we binned available templates into groups with 15–19%, 20–24%, 25–29%, and 30–39% sequence identity (there were not enough templates in the 30–39% range to split into two bins). We then identified three receptors with at least five templates in each identity group and performed multiple-template homology modeling with each set of five templates. The results, shown in Fig 6, find that overall, the TM Region accuracy is unaffected by the use of templates down to 20% sequence identity. The same trend held true for the full model accuracy seeing a dramatic rise in RMSD with templates lower than 20% identity. ECL2 was the most sensitive region where accuracy drops sharply when lower identity templates are used. This is to be expected as structural conservation of the ECL2 loop appears to be family dependent and using lower template structures requires moving to more divergent receptors. Taken together, we suggest that templates down to 20% identity yield accurate models, particularly within the TM region which is important for understanding the ligand recognition site.

### Development of database for all human non-odorant GPCRs

By effectively pushing the lower threshold to 20% sequence identity, we can now predict models for the remaining GPCRs deemed druggable. To this effect, we identified the best templates (as of June 2020) by identity for the entire set of non-olfactory human GPCRs (S2 Fig). Out of 397 receptors, 73 have at least one structure determined. This provides 81 receptors with a template with sequence identity above 40%, the previous threshold for accurate modeling. However, the number of receptors with a template between 20 and 40% is 201. Only 41 receptors (or 10% of the receptor family) remain with sequence identities less than 20% and thus expected lower accuracy in their models. We therefore applied our methodology to the entire family of non-odorant Class A, B, C, and F GPCRs to create a model database available at www.rosettagpcr.org. This is, to the best of our knowledge, the only Rosetta-based GPCR server available, which distinguishes it from the many Modeller-based servers. While it is true that the Robetta server exists for general homology modeling, it is not designed with a membrane potential and it relies on automatic alignment methods, which we show to be insufficient for low identity templates. The advantage of using a Rosetta-based modeling server is that the models can be used directly in other Rosetta applications like ligand docking, protein-protein docking, and design. Switching models between different programs can result in problems due to intrinsic differences in energy functions. Currently all models are in the inactive state though will likely expand to active state models as more active state structures are determined by cryo-electron microscopy. Additionally, it has been shown that co-modeling ligands into homology models can improve the accuracy of a given model with respect to an experimental structure. We have preliminary data suggesting that to be true for RosettaGPCR as well (S3 Fig). However, for the purpose of modeling all receptors, the chemical and conformational space is too large a problem to be tackled by mass modeling and is best suited for individual target application. To this end, we provide all input files needed so that a given receptor model can be improved with target-specific information such as ligand co-modeling.

## Discussion

Accurate modeling of targets from low-identity templates is a challenge for computational biology. GPCRs represent an unmet need in this area as most GPCRs remain structurally uncharacterized and possess identities on average below 40% to other receptors. Many of these proteins already have FDA approved drugs targeting them [3], but a deep understanding of the molecular basis of drug intervention is lacking. Further, about a third of these receptors are classified as orphan receptors because the endogenous ligand has not been identified [8]. A structural perspective of the ligand binding pocket may help shed light on this group of receptors. We have outlined here key considerations for homology modeling of proteins with low-sequence identity to templates using GPCRs as a focus. Inputs are critical to the success of any homology modeling program and optimizing them should be a priority to any new modeling campaign. We show that improved alignments, incorporation of peptide fragments to overcome template inaccuracies, and limiting the set of templates to the most relevant can allow for accurate modeling of structures with templates below 40% identity, and subsequently apply this method to all non-odorant GPCRs to produce the RosettaGPCR database. However, we want to stress that the methodology is applicable to any homology modeling programs. We have provided all necessary inputs to generate our database with the hope that a new user will adapt the protocol to their specific test case and expert knowledge, including ligand information, mutational analysis, or new template availability.

## Supporting information

S1 Table List of Receptors in Benchmark. Receptor name and the corresponding PDB ID that was used for accuracy measurements.
(TIF)

S2 Table List of Templates for Each Target Ranked by Sequence Identity. Yellow highlighted templates were not used for general modeling because they have sequence identities greater than 40%. Bolded templates were used for single-template high identity modeling to compare to previous benchmark.
(TIF)

S1 Fig Alignment of receptor sequences. The alignment for all 34 receptors is shown using Aline [45]. Identical and highly conserved residues are color-coded for easy identification. Alignment available at www.rosettagpcr.org.
(TIF)

S2 Fig Percent Identify of Best Available Template for Every Non-Odorant Human GPCR. For each receptor in the human genome, the best template was identified in the PDB. The sequence identity of the best available is plotted. Most templates cross the 20% threshold identified as critical for accurate modeling. The previous threshold of 40% identity is highlighted in red, and the new 20% identity threshold is highlighted in black.
(TIF)

S3 Fig Effect of Incorporation of a Ligand on Model Accuracy. One hundred models of the D3 receptor was modeled either with multiple low identity templates (red) or with a single high identity template (green) and the full model RMSDs are plotted as violin plots. As expected, the high identity template yields higher accuracy models on average. Incorporation of a ligand during the modeling process further improve the accuracy compared to the apo state for both multiple low identity templates (cyan) and a single high identity template (purple).
(TIF)

S1 Protocol Protocol Capture for Homology Modeling from Low Identity Templates. Step-by-step guide to build models using this pipeline. Input data and scripts available at www.github.com/benderb1/rosettagpcr.
(DOCX)
