# Supplementary figures and images for: Improving homology modeling from low-sequence identity templates in Rosetta: A case study in GPCRs
Source: PLoS Comput Biol. 2020 Oct 28;16(10):e1007597. doi: 10.1371/journal.pcbi.1007597 (PMC7652349; doi:10.1371/journal.pcbi.1007597)

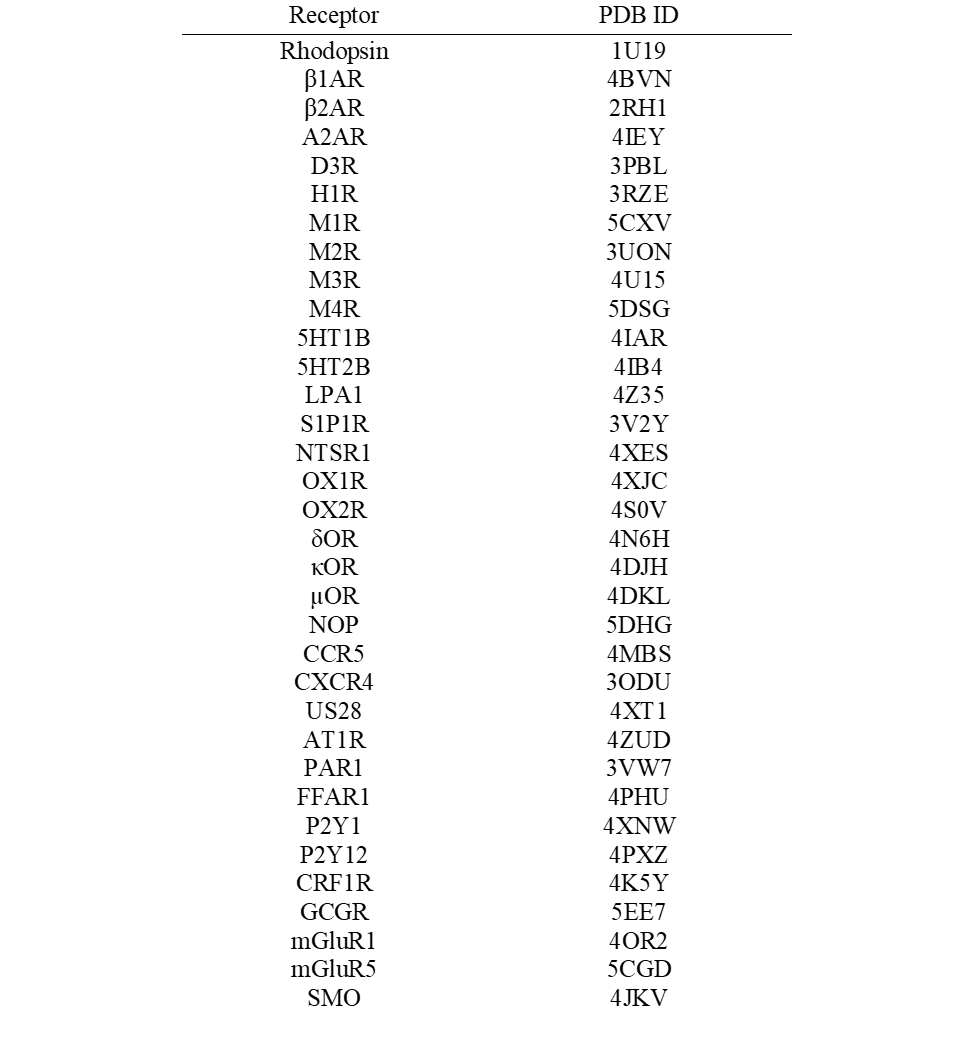

Supplement: S1 Table — Receptor name and the corresponding PDB ID that was used for accuracy measurements. (TIF) [file pcbi.1007597.s001.tif]

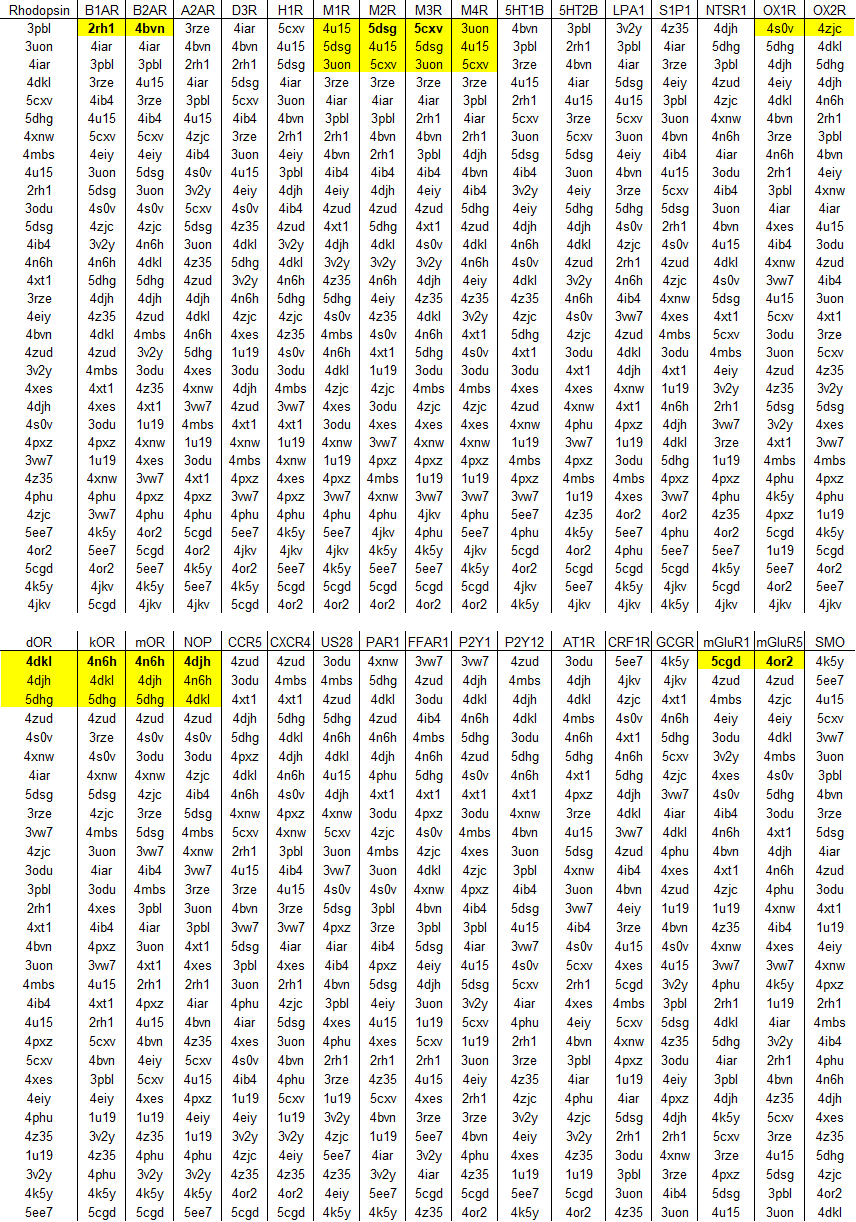

Supplement: S2 Table — Yellow highlighted templates were not used for general modeling because they have sequence identities greater than 40%. Bolded templates were used for single-template high identity modeling to compare to previous benchmark. (TIF) [file pcbi.1007597.s002.tif]

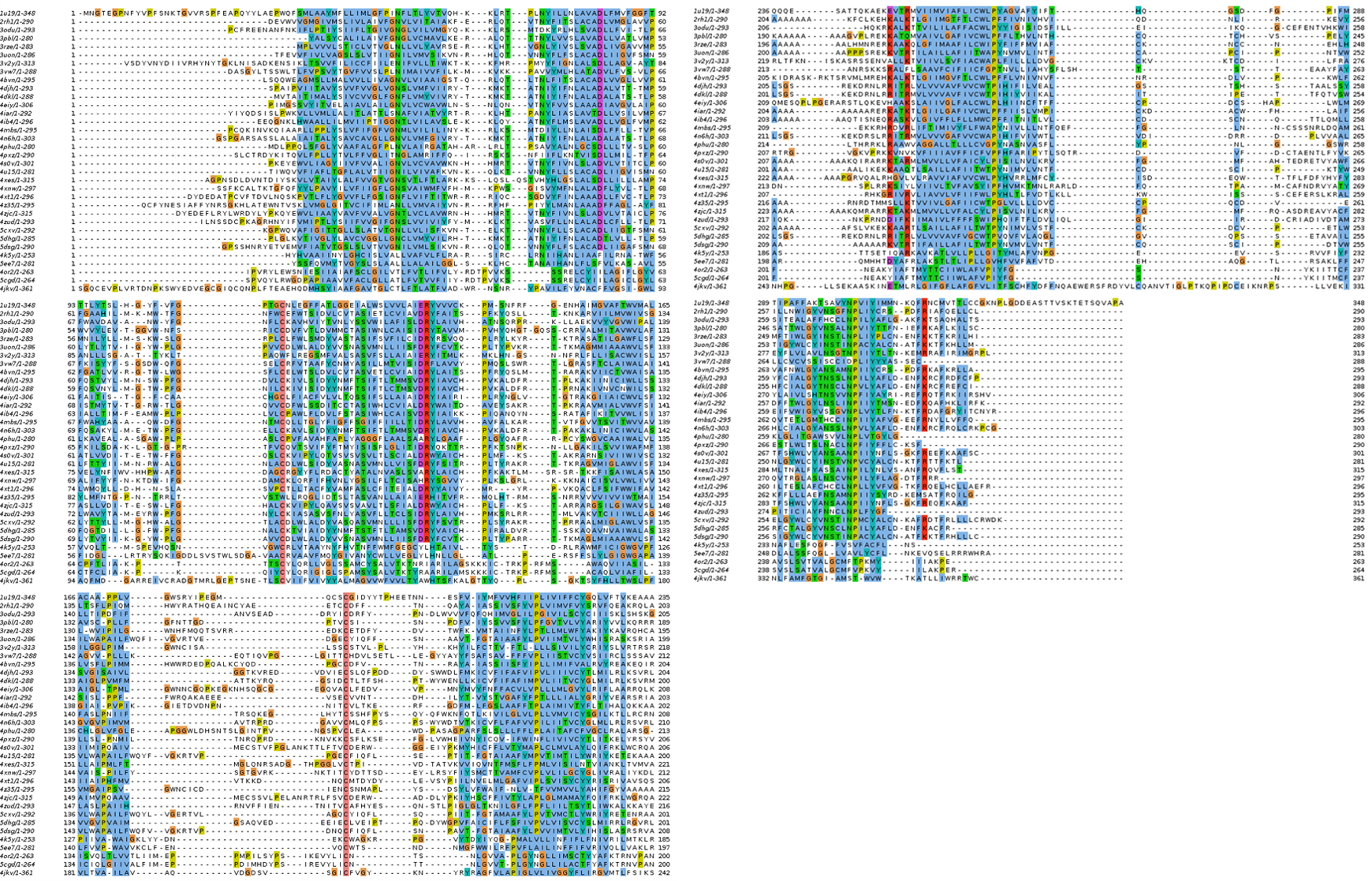

Supplement: S1 Fig — The alignment for all 34 receptors is shown using Aline [45]. Identical and highly conserved residues are color-coded for easy identification. Alignment available at www.rosettagpcr.org. (TIF) [file pcbi.1007597.s003.tif]

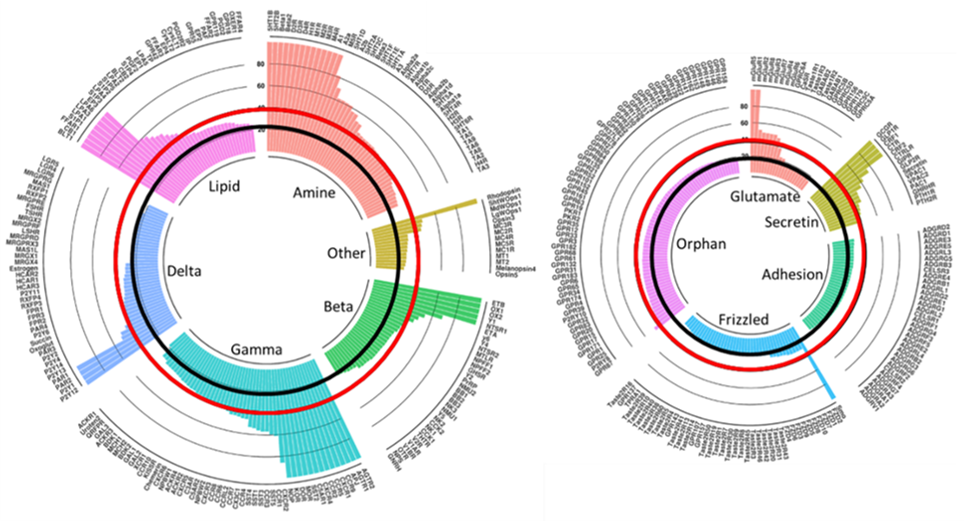

Supplement: S2 Fig — For each receptor in the human genome, the best template was identified in the PDB. The sequence identity of the best available is plotted. Most templates cross the 20% threshold identified as critical for accurate modeling. The previous threshold of 40% identity is highlighted in red, and the new 20% identity threshold is highlighted in black. (TIF) [file pcbi.1007597.s004.tif]

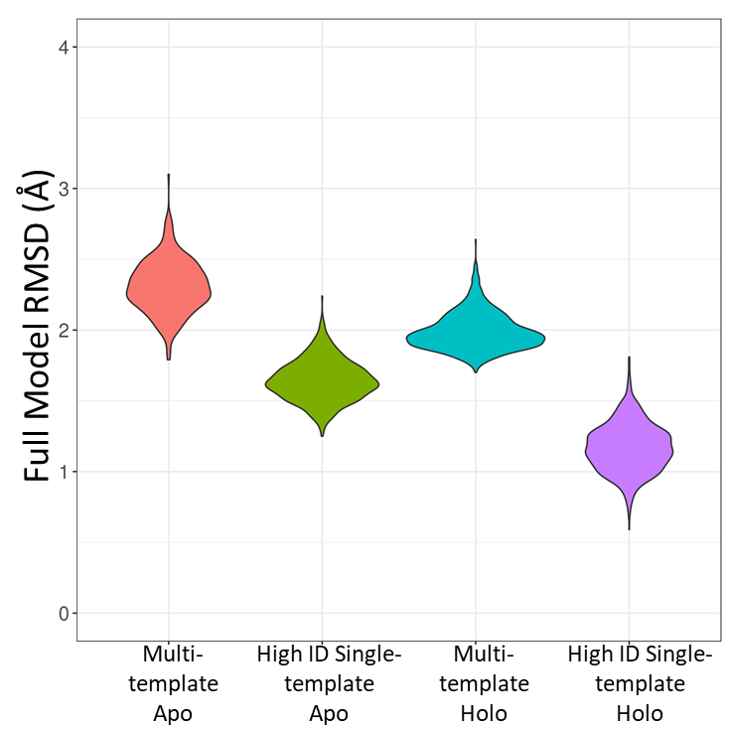

Supplement: S3 Fig — One hundred models of the D3 receptor was modeled either with multiple low identity templates (red) or with a single high identity template (green) and the full model RMSDs are plotted as violin plots. As expected, the high identity template yields higher accuracy models on average. Incorporation of a ligand during the modeling process further improve the accuracy compared to the apo state for both multiple low identity templates (cyan) and a single high identity template (purple). (TIF) [file pcbi.1007597.s005.tif]
