# Supplementary material for: Improving homology modeling from low-sequence identity templates in Rosetta: A case study in GPCRs
Source: PLoS Comput Biol. 2020 Oct 28;16(10):e1007597. doi: 10.1371/journal.pcbi.1007597 (PMC7652349; doi:10.1371/journal.pcbi.1007597)
Supplement: S1 Protocol — Step-by-step guide to build models using this pipeline. Input data and scripts available at www.github.com/benderb1/rosettagpcr. (DOCX) [file pcbi.1007597.s006.docx]

Protocol for Homology Modeling from Low Identity Templates

File availability:

- Model database: [www.rosettagpcr.org](http://www.rosettagpcr.org)
- Scripts and input data: [www.github.com/benderb1/rosettagpcr](http://www.github.com/benderb1/rosettagpcr)

Necessary Inputs:

- Target fasta (/PATH/TO/rosettagpcr/class_a/{target}/target.fasta)
- Template pdbs (find new or pull from /PATH/TO/rosettagpcr/class_a/templates/)
- Alignment file (make or lives in /PATH/TO/rosettagpcr/class_a/{target}/alignment.fasta)

Step 1: Pick templates

- The templates that are present in /PATH/TO/rosettagpcr/class_a/{target}/alignment.fasta are the best templates by identity as of June 2020, if better templates have since been released or you want to change templates for a reason specific to your target do the following
  - Search template structures by identity to target
  - Also consider whether template is in the same receptor family (i.e. don’t use a class C gpcr to model a Class A receptor)
  - Consider ligand similarity (i.e. use peptide-binding receptors to model target peptide-binding receptor)
  - Want up to 5 templates
    - If available templates are >50% ID, need one template
    - If less than 50% ID, consider multiple templates
    - Below 20% ID modeling is possible but likely suffers accuracy

Step 2: Prepare templates

- First start in /PATH/TO/rosettagpcr/class_a/templates/ to look for already prepared and aligned template pdbs, otherwise do the following
- Remove fusion proteins and non-ligand HETATMs (i.e. cholesterols/PEGs/etc.)
- Align templates in space
  - Suggestion: select the TM helices of one template and align other templates to just this region in PyMol or Chimera.
- Save aligned template pdbs

Step 3: Prepare alignment file

- Start with /PATH/TO/rosettagpcr/class_a/{target}/alignment.fasta if you are happy with the templates that were preselected, otherwise:
- Go to /PATH/TO/rosettagpcr/class_a/ and find alignment.fasta
  - This is a master alignment of all GPCRs
- Pull out the fastas for your target and templates
  - There are a number of template pdbs already included in this alignment file (i.e. 6ndd_) corresponding to the templates that live in /PATH/TO/rosettagpcr/class_a/templates/
  - Use these template alignments since they will already have accounted for non-resolved residues, truncated termini, and thermostabilizing mutations
  - **If your template does not have a provided crystallographic alignment**, the template alignment will need to be checked for non-resolved residues, truncated termini, and thermostabilizing mutations
- Adjust alignment according to the structural alignment of the templates
  - For most receptors this is already done but it always critical to check
- When alignment is optimized, save alignment file such that the target sequence is first and then template sequences are ordered from best template to worst

Step 4: Setup RosettaCM

- With target.fasta, alignment.fasta, and (aligned) template pdbs use the following command to prepare a directory for modeling
  - python /PATH/TO/rosettagpcr/scripts/setup_RosettaCM.py --fasta target.fasta --alignment alignment.fasta --templates {template pdbs}
  - Other options exist for advanced use with the --help option
- This script creates a new directory rosetta_cm, threads your target sequence onto the template pdbs, generates the rosetta_cm.xml and flags files that are needed to run RosettaCM

Step 5: Prepare span and disulfide files

- Change into the rosetta_cm directory
- Make disulf.txt
  - Identify the sequence position of cysteines involved in disulfide formation (i.e. TM3 and ECL2)
  - Make a file “disulf.txt” that contains these each pair of residue positions separated by whitespace, i.e.
    - 93 151
- Make span file
  - The Rosetta score function needs to be told which residues live in the membrane, this is the purpose of the span file
  - Go to <http://octopus.cbr.su.se/>
  - Copy and paste your target sequence into the text box and hit submit
  - On the results page, click on link to “OCTOPUS topology file (txt)”
  - Copy the entire page into a text file called octopus.txt
  - Run script to convert octopus format to Rosetta format
    - /PATH/TO/Rosetta/tools/membrane_tools/octopus2span.pl octopus.txt > span.txt
  - Format should look like:

TM region….

7 325 #### number of TM spans and number of residues

antiparallel

n2c

7 lines with 4 numbers each corresponding to start and end position of each span

- - **IF ADDING A LIGAND**, add 1 residue to the second number in line 2
    - So in the above example 325 becomes 326

Step 6: Check that all inputs are present and run homology modeling

- In the rosetta_cm directory there should be
  - rosetta_cm.xml
  - flags
  - span.txt
  - disulf.txt
  - threaded template pdbs
  - output directory
- If everything is present run this command to generate a single model
  - /PATH/TO/Rosetta/main/source/bin/rosetta_scripts.default.linuxgccrelease @flags -database /PATH/TO/Rosetta/main/database -nstruct 1
- Output models live in the output directory

Optional: Modeling with a Ligand Present

If you want to add a ligand in your model, a few extra steps are needed

- Using a crystallized ligand from a template
  - If the template is aligned with the other templates, save the ligand coordinates as its own pdb file
  - Convert the ligand pdb to mol2 file and add hydrogen atoms
    - /PATH/TO/openbabel/current/bin/obabel -ipdb xtal-lig.pdb -omol2 -O xtal-lig.mol2 -h -p 7.4
  - Convert the mol2 file into a Rosetta readable file
    - /PATH/TO/Rosetta/main/source/scripts/python/public/molfile_to_params.py xtal-lig.mol2 --keep-names --clobber --extra_torsion_output --centroid -p lig -n lig
    - Outputs should be
      - lig_0001.fa.pdb
      - lig_0001.cen.pdb
      - lig.fa.params
      - lig.cen.params
      - lig.fa.tors
      - lig.cen.tors
  - Cat the lig.pdb coordinates to the **threaded** pdbs in the rosetta_cm directory
    - cat lig_0001.fa.pdb >> template_thread.pdb
  - In the flags file add:
    - -extra_res_cen lig.cen.params
    - -extra_res_fa  lig.fa.params
    - -extra_improper_file lig.cen.tors
  - In the span file:
    - Update the total length of the protein to have one additional residue
    - I.e: Original
      - 7 278
    - Updated
      - 7 279
  - In the rosetta_cm.xml file
    - Find the line that starts “Hybridize”, the end of this line should be the disulfide file path
    - Between the end of the disulfide file path and before the “>” symbol add the following:
      - add_hetatm=”1”
  - Run modeling using the above provided command line
- Using an analog of a crystallized ligand from a template
  - Align the analog to the crystallized ligand
  - Save the coordinates of the analog as xtal-lig.pdb
  - Use above pipeline
- Using a fully unrelated ligand to any of the templates
  - Consider docking against a threaded template or some other method to get a reasonable starting pose (i.e. does it match known experimental data)
  - Use the coordinates of the docked pose in the above pipeline
